# Exploration of Reduced Mitochondrial Content–Associated Gene Signature and Immunocyte Infiltration in Colon Adenocarcinoma by an Integrated Bioinformatic Analysis

**DOI:** 10.3389/fgene.2022.832331

**Published:** 2022-04-08

**Authors:** Jinlin Kang, Na li, Fen Wang, Yan Wei, Yangyang Zeng, Qifan Luo, Xuehua Sun, Hui Xu, Jin Peng, Fuxiang Zhou

**Affiliations:** ^1^ Department of Radiation and Medical Oncology, Zhongnan Hospital Wuhan University, Wuhan, China; ^2^ Hubei Province Key Laboratory of Tumor Biological Behaviors, Wuhan, China; ^3^ Hubei Cancer Clinical Study Center, Wuhan, China; ^4^ Renmin Hospital of Wuhan University, Wuhan, China

**Keywords:** mitochondiral DNA, Colon Cancer, Immunocyte infiltration, Bioinformatic analysis, Gene signature

## Abstract

**Purpose:** Mitochondrial dysfunction refers to cancer immune evasion. A novel 7-gene prognostic signature related to the mitochondrial DNA copy number was utilized to evaluate the immunocyte infiltration in colon cancer according to the risk scores and to predict the survival for colon cancer.

**Experimental design:** We performed an integrated bioinformatic analysis to analyze transcriptome profiling of the EB-treated mitochondrial DNA–defected NCM460 cell line with differentially expressed genes between tumor and normal tissues of COAD in TCGA. The LASSO analysis was utilized to establish a prognostic signature. ESTIMATE and CIBERSORT validated the differences of immunocyte infiltration between colon cancer patients with high- and low-risk scores.

**Results:** Our study identified a 7-gene prognostic signature (*LRRN2*, *ANKLE1*, *GPRASP1*, *PRAME*, *TCF7L1*, *RAB6B*, and *CALB2*). Patients with colon cancer were split into the high- and low-risk group by the risk scores in TCGA (training cohort: HR = 2.50 *p* < 0.0001) and GSE39582 (validation cohort: HR = 1.43 *p* < 0.05). ESTIMATE and CIBERSORT revealed diverseness of immune infiltration in the two groups, especially downregulated T-cell infiltration in the patients with high-risk scores. Finally, we validated the colon patients with a low expression of the mitochondrial number biomarker TFAM had less CD3^+^ and CD8^+^ T-cell infiltration in clinical specimens.

**Conclusion:** An mtDNA copy number-related 7-gene prognostic signature was investigated and evaluated, which may help to predict the prognosis of colon cancer patients and to guide clinical immunotherapy *via* immunocyte infiltration evaluation.

## Introduction

Colon cancer is the fourth most common malignant tumor, which caused approximately 247,563 deaths in China in 2018 (Feng et al., 2019). The incidence and mortality of colorectal cancer increased rapidly with the economic development in recent years (Chen et al., 2016). Molecular testing plays an increasingly important role in estimating prognosis and deciding the best therapy for colon cancer patients with worse prognosis (Van Cutsem et al., 2016). We focused on mitochondrial dysfunction, which has been proved to play diverse roles in cancer metabolism, immune response, and cell signaling pathways (Hutson et al., 1988; West et al., 2011; Wallace, 2012). Mitochondria have their own 16-kilobase mitochondrial genome independent of the nucleus genome, which encodes two rRNAs, 22 tRNAs, and 13 polypeptides. The alterations of mitochondrial DNA (mtDNA) contribute to aberrant mitochondrial respiration, metabolism, and other cellular functions (Yu, 2011). Metabolism alteration has been commonly acknowledged as one of the hallmarks of cancer, which is associated with tumorigenesis, tumor microenvironment, cell progression, immune infiltration, and treatment response (Elia and Fendt, 2016; Pavlova and Thompson, 2016; Lunt and Fendt, 2017). Notably, increasing evidence demonstrated the relationship between immune cell infiltration and dysregulated metabolic pathways (Peteret al., 2017; Li et al., 2019). The alterations of metabolism promoted reprogramming of the tumor microenvironment (TME), herein enhanced immunostimulation (Cheng et al., 2014; Arts et al., 2016).

Following the inspiring success in melanoma and lung cancer, immunotherapy has been applied in the treatment of colon cancer since 2017. However, current immune checkpoint inhibitors (ICIs) merely showed less effect in several restricted subtypes of colon cancer (Ganesh et al., 2019). Accumulating evidence suggests that less infiltration of immunocytes showed the contribution of immune resistance (Herrera et al., 2013). The immune system recognizes itself through binding T-cell receptors (TCRs) to the human leukocyte antigen (HLA). This process is modulated by co-stimulatory factors named immune checkpoints, such as programmed cell death 1 (PD1) and cytotoxic T-lymphocyte antigen 4 (CTLA4) (Sharma and Allison, 2015). However, there were only few studies that clarified the relationship between mtDNA copy number reduction and tumor immunocyte infiltration. The value of aforementioned seven signature genes in predicting the immunocyte infiltration in colon cancer was assessed. Subsequently, the results were validated in GSE39582 (*n* = 550) and estimated using the ESTIMATE algorithm. Taken together, we utilized integrated bioinformatic analysis to select seven significant genes positively related to mitochondrial content reduction and confirmed their potential values in colon cancer to predict the prognosis and evaluate immune infiltration.

## Material and Methods

### Data Acquisition

HTSeq-FPKM workflow type transcriptome data and clinical data of colon adenocarcinoma (COAD) were obtained from TCGA (https://portal.gdc.cancer.gov). GEO datasets (GSE39582) were obtained from GEO (https://www.ncbi.nlm.nih.gov/geo/). Only those patients whose overall survival days were more than 30 days were collected.

### Cell Lines and Culture Condition

The human immortalized colon cell line NCM460 was acquired from Procell Company. (Wuhan, China). The cells were cultured in Dulbecco’s modified Eagle medium (DMEM; Thermo Fisher, United States) with 10% fetal bovine serum (Gibco, United States) and 1% pen-strep (100 U/ml penicillin and 100 mg/ml streptomycin) (Gibco, United States). The mtDNA copy number knockdown was induced by maintaining in 50 ng/ml ethidium bromide (EB) with 1 mM sodium pyruvate (Sigma-Aldrich, St. Louis, MO, United States) and 50 μg/ml uridine (Sigma-Aldrich, St. Louis, MO, United States) ethidium bromide (EB) maintained for over 4 months.

### RNA-Seq and Data Analysis

Total RNA was extracted from NCM460 using TRIzol. RNA samples were taken from EB-treated NCM460 and normal NCM460 cells; RNA transcriptome analysis was conducted by Seqhealth Technology Company (Wuhan, China).

### Differentially Expressed Gene Selection

The transcriptome data of NCM460 and EB-treated NCM460 cells, COAD in TCGA dataset, and GSE39582 were extracted for further analysis. Differences between tumor tissue and adjacent normal tissue were collected using the limma package. |logFC| more than 0.5 and the false discovery rate (FDR) less than 0.05 were set down as the cut-off standard.

### Functional Enrichment Analysis

The functional analysis of DEGs was implemented using clusterProfiler.

### PPI Network Analysis

The analysis of the PPI network with the confidence score >0.9 was performed using the Metascape website (https://metascape.org) and Cytoscape. In order to visualize the PPI network and screen out valuable hub genes, we utilized the MCODE plug‐in of Cytoscape software with the degree as 5, node score as 0.2, k‐core as 2, and maximum depth as 100 for cut‐off criteria

### Estimation of Immune Cell Proportions and Infiltration

The correlation scores of individual samples were evaluated using the ESTIMATE algorithm. The expressions of the HLA family genes, CTLA4, and 22 immune-related subtypes were analyzed between the low-risk and high-risk groups using the limma and CIBERSORT package.

### Survival Analysis

The Kaplan–Meier survival analysis was performed to assess the correlation between the patient survival probability and different groups, which were characterized by the risk score related to the 7-gene feature expression in TCGA calculated by LASSO. The results were validated in GSE39582 (*n* = 550).

### Identification of Prognostic Characteristics of Immune-Related Genes

All of the 475 DEGs (*p* < 0.05) were screened by overlapping transcriptome DEGs from tumor vs. adjacent normal tissue and DEGS from NCM460 cells treated with ethidium bromide (EB) or not. Among them, 12 genes were assayed by LASSO Cox analysis with 10-round cross-validation. Stepwise multiple Cox regression analysis was performed to select and optimize prognostic features. Univariate and multivariate Cox regression analyses assessed the independence of the prognostic signature from clinical factors. Time-dependent ROC analysis was performed using the survival package. Nomogram and calibration curves were accomplished using the rms package.

### Immunohistochemistry

Patients pathologically diagnosed by biopsy in Zhongnan hospital, Wuhan University, in the past 3 years were included in our research. The paraffin sections of surgical tissues of 39 colon cancer patients were used for the immunohistochemical analyses. These sections were put into xylene, absolute ethanol, 85% alcohol, and 75% alcohol for deparaffinzation and rehydration. The tissue sections were filled with citric acid (PH6.0) in a microwave oven and heated on medium power for antigen retrieval. The sections were put into 3% hydrogen peroxide and incubated at 4°C in darkness. To cover the tissues, the sections were sealed for 30 min in 3%BSA at 4°C. The sections were placed flat in a wet box and incubated with primary antibodies against transcription factor A (TFAM, Proteintech), CD3 (Servicebio GB13014), and CD8 (Servicebio GB13429 overnight at 4°C. The samples were then incubated with the secondary antibody (HRP-labeled) for 50 min. The samples were visualized and counterstained with DAB and hematoxylin and then dehydrated in ethanol and cleared in xylene. The sections were placed and shaken on the decolorization shaker three times completely for 15 min in PBS(PH7.4) after each step. The intensities of CD3, CD8, and TFAM were visualized by Image-Pro Plus 6.0 and were analyzed by Halo (India labs, United States).

### Quantitative Reverse Transcription PCR

Total RNA of SW480 cells was extracted using the RNeasy Mini Kit, while reverse transcription was performed using the Prime Script RT Reagent Kit. SYBR Premix Ex Taq (Aidlab Biotechnologies) was utilized for real-time PCR at an ABI Prism 7900 instrument (Applied Biosystems).

The primer sequences used in this research are as follows:

LRRN2: F CGA​GGC​TAC​CAC​TGT​GGA​C

LRRN2: R GGG​CAT​CCG​AAA​AGC​TGT​TC

ANKLE1: F GAC​CCC​AAC​GCT​CGA​TCT​G

ANKLE1: R TCG​GGC​TCC​TGA​GTC​TCT​G

GPRASP1: F AGG​AGG​AGA​CCA​ATA​TGG​GGT

GPRASP1: R GGA​CCT​AGA​CAT​GGT​ATT​AGC​CT

PRAME: F TGG​AAT​TAA​CTT​GTG​GCA​ACC​T

PRAME: R TCT​GAC​AGC​CCT​CTA​ACA​CGA

TCF7L1: F TCG​TCC​CTG​GTC​AAC​GAG​T

TCF7L1: R ACT​TCG​GCG​AAA​TAG​TCC​CG

RAB6B: F TGT​ACG​ACA​GCT​TCG​ACA​ACA

RAB6B: R CTG​CGG​AAC​CTC​TCC​TGA​C

CALB2: F ACT​TTG​ACG​CAG​ACG​GAA​ATG

CALB2: R GAA​GTT​CTC​TTC​GGT​TGG​CAG

*β*-Actin: F CAT​GTA​CGT​TGC​TAT​CCA​GGC

*β*-Actin: R CTC​CTT​AAT​GTC​ACG​CAC​GAT

### Statistical Analysis Method

Statistical analysis was performed in R version 4.0.3. The correlations of protein expression in pathological data were analyzed using prism 7.

## Results

### Identification and Functional Analysis of DEGs

**FIGURE 1:**
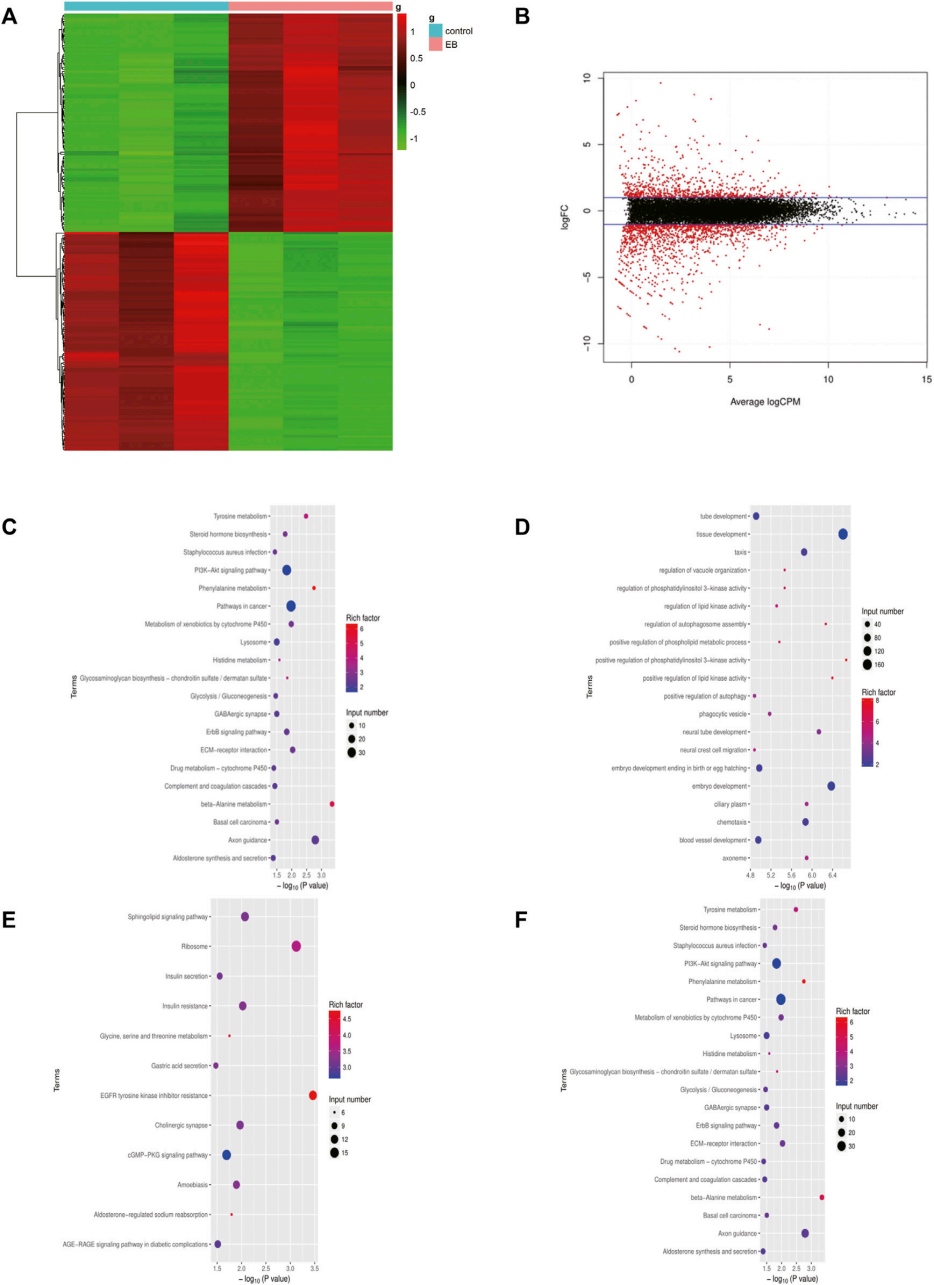
DEGs identified in the EB-treated NCM460 cell line. **(A)** Heatmap of the top 200 DEGs by |logFC|. The color from blue to red indicates low to high expression level. **(B)** Volcano map between EB-treated and control groups of the NCM460 cell line. **(C,D)** Enriched terms in GO analysis of DEGs in EB-treated NCM460, respectively. **(E,F)** Enriched terms in KEGG analysis of DEGs in EB-treated NCM460 DEGs, respectively; DEGS means differentially expressed genes; logFC means log fold change.

The gene expression profile of the mtDNA-reduced NCM460 cell line, which was derived from the normal human colon mucosal epithelium, was analyzed using the “limma” package. A total of 2075 DEGs were screened, of which 794 genes were upregulated while 1281 genes were downregulated. The results are shown in Figures 1A,B. To first realize the function of DEGs of EtBr-treated colon cells, the alternated DEGs were transmitted to the “clusterProfiler” for functional annotations. GO and KEGG term enrichment analyses showed that the upregulated DEGs were fairly enriched, as shown in (Figures 1C–F). Furthermore, we found that DEGs in the biological process (BP) group were enriched in “signal transduction, cell adhesion, oxidation–reduction process, positive regulation of GTPase activity, and positive regulation of cell proliferation” based on the results of GO analysis of all DEGs. In the cellular composition (CC) group, all DEGs were associated with the “integral component of membrane, plasma membrane, cytosol, extracellular exosome, and integral component of plasma membrane.” In addition, the terms “calcium ion binding, receptor binding, kinase activity, heparin binding, and oxidoreductase activity” were enriched in the molecular function (MF) module. These apparently elevated terms of DEGs supported the notion that they functioned in carcinogenesis and progression for colon cancer in patients with mtDNA content reduction.

### Intersection of mtDNA Content Reduction–Associated DEGs and Prognostic-Related DEGs

**FIGURE 2:**
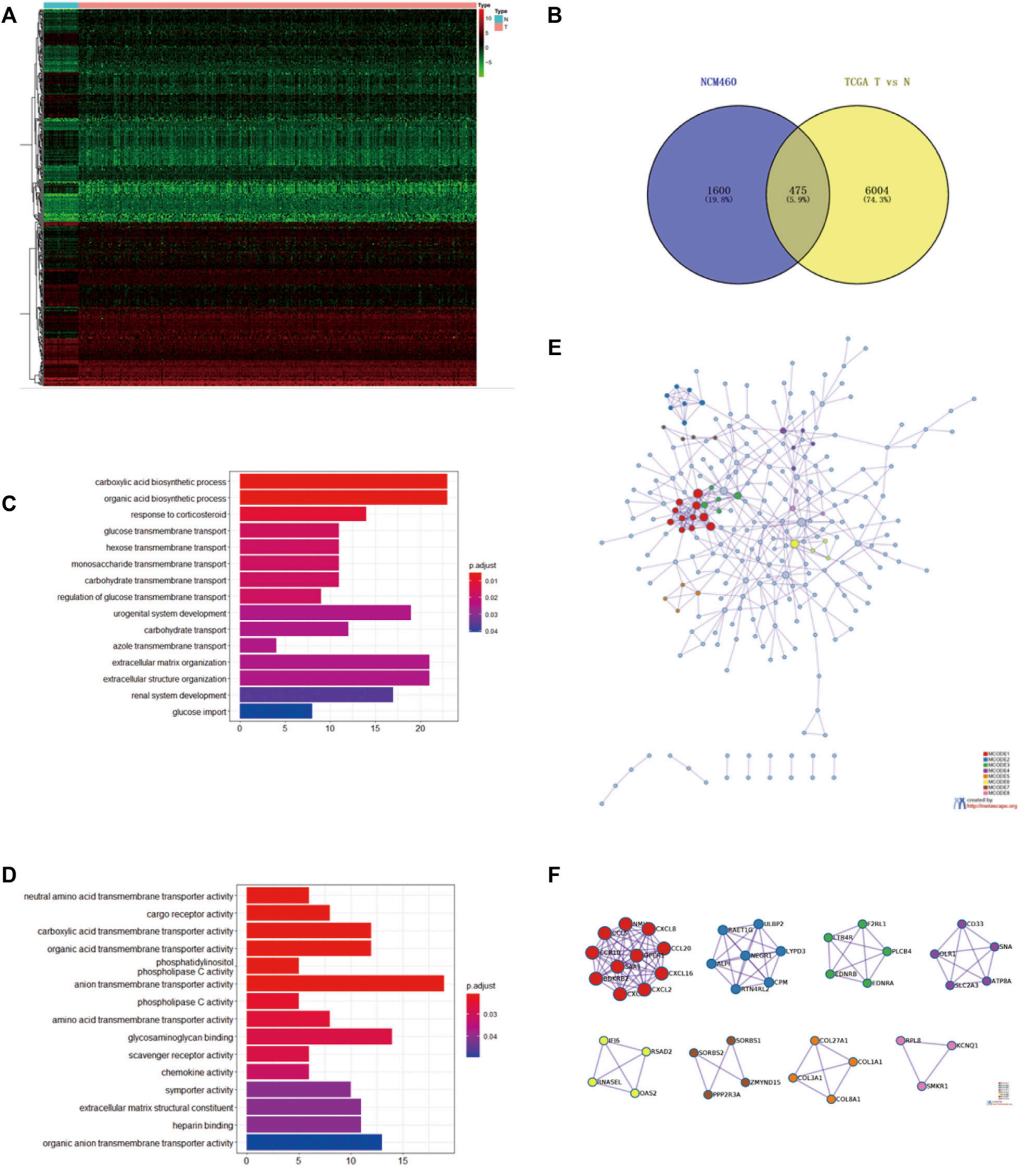
Intersection of the mtDNA-related gene and prognostic gene in TCGA **(A)**. Heatmap of the top 200 DEGs identified between tumor and compared normal tissues from COAD in TCGA based on the value of |logFC| **(B)**. Intersection of 475 common DEGs with the prognostic value in TCGA and EB-treated NCM460 using the Venn diagram. Top three hub modules were classified in MODE. Red circles represented upregulated genes, while blue circles represented downregulated genes. **(C,D)** Heatmap of GO enriched terms and biological process (BP) GO enriched terms. **(E)** PPI network of the common 475 genes calculated by STRING software from the Metascape website **(F)** Top eight modules of the PPI network; GO, gene ontology; PPI, protein–protein interaction [Color figure can be viewed at wileyonlinelibrary.com].

Comparing the transcriptome of NCM460 and the EtBr-treated NCM460 cell line, 2075 DEGs were identified, which were composed of 794 upregulated genes and 1281 downregulated genes. We selected 475 DEGs using the Venn diagram of prognostic genes in TCGA datasets and EB-treated NCM460 DEGs (Figures 2A,B). Functional analysis was performed *via* clusterProfiler in R. The top GO terms included the carboxylic acid biosynthetic process and organic acid biosynthetic process. The network of GO enriched terms is displayed in Figures 2C,D. Anion transmembrane transporter activity and glycosaminoglycan binding are enriched in the biological processes (BPs). Interestingly, there are no items enriched in the cellular composition (CC), and the top 15 terms are the same in all GO and MF (Figure 2C). It seemed DEGs are mostly correlated with the biosynthetic process and transmembrane transport. In addition, 475 DEGs were analyzed using the Search Tool for the Retrieval of Interacting Genes database (STRING) and performed to formulate the protein–protein interaction (PPI) network (Figure 2E). Central modules were instituted using Molecular Complex Detection (MCODE) (Figure 2F). The results gained from STRING showed that the PPI network of DEGs consisted of 252 nodes and 444 edges. The top 30 distinguished proteins were classified as hub genes, which might play vital roles in the mtDNA content reduction related to tumor initiation through PPI analysis. A total of eight clusters were generated in MCODE, and the top three clusters were selected as hub modules by the scores evaluated in MCODE (Supplementary Table S2). The 11 genes in MCODE1 (*CCR10*, *CXCL16*, *CXCL2*, *CXCL3*, *CXCL8*, *GPER1*, *NMU*, *SAA1*, *BDKRB2*, *CCL20*, and *CCL5*) were associated with cell chemotaxis, which induced the directional migration of cells including cancer cells and immune cells.

### Construction and Validation of a 7-Gene Prognostic Signature

We implemented a prognostic analysis of 475 DEGs by the overlap of prognostic genes in TCGA datasets and NCM460 EB-related DEGs. The significant prognostic factors revealed by univariate Cox proportional hazards regression (*p* < 0.001) were SNAP25, LRRN2, ANKLE1, GPRASP1, CD37, PRAME, PDZD4, TCF7L1, RAB6B, CALB2, DUSP9, and SUSD5 (Supplementary Table S1).From the least absolute shrinkage and selection operator (LASSO) Cox regression analysis, 12 genes were filtered for stepwise multivariate Cox regression analysis (Supplementary Table S1). Finally, seven key genes (*LRRN2*, *ANKLE1*, *GPRASP1*, *PRAME*, *TCF7L1*, *RAB6B*, and *CALB2*) were generated to manufacture the prognostic signature: risk score = 0.231*LRRN2+ 0.448*ANKLE1+0.086*GPRASP1+ 0.118*PRAME+ 0.104*TCF7L1+ 0.171*RAB6B+ 0.049*CALB2. The distribution of the risk score, survival status, and gene expression profiles between the two groups in TCGA and GSE39582 are displayed in Fig.4.

**FIGURE 3:**
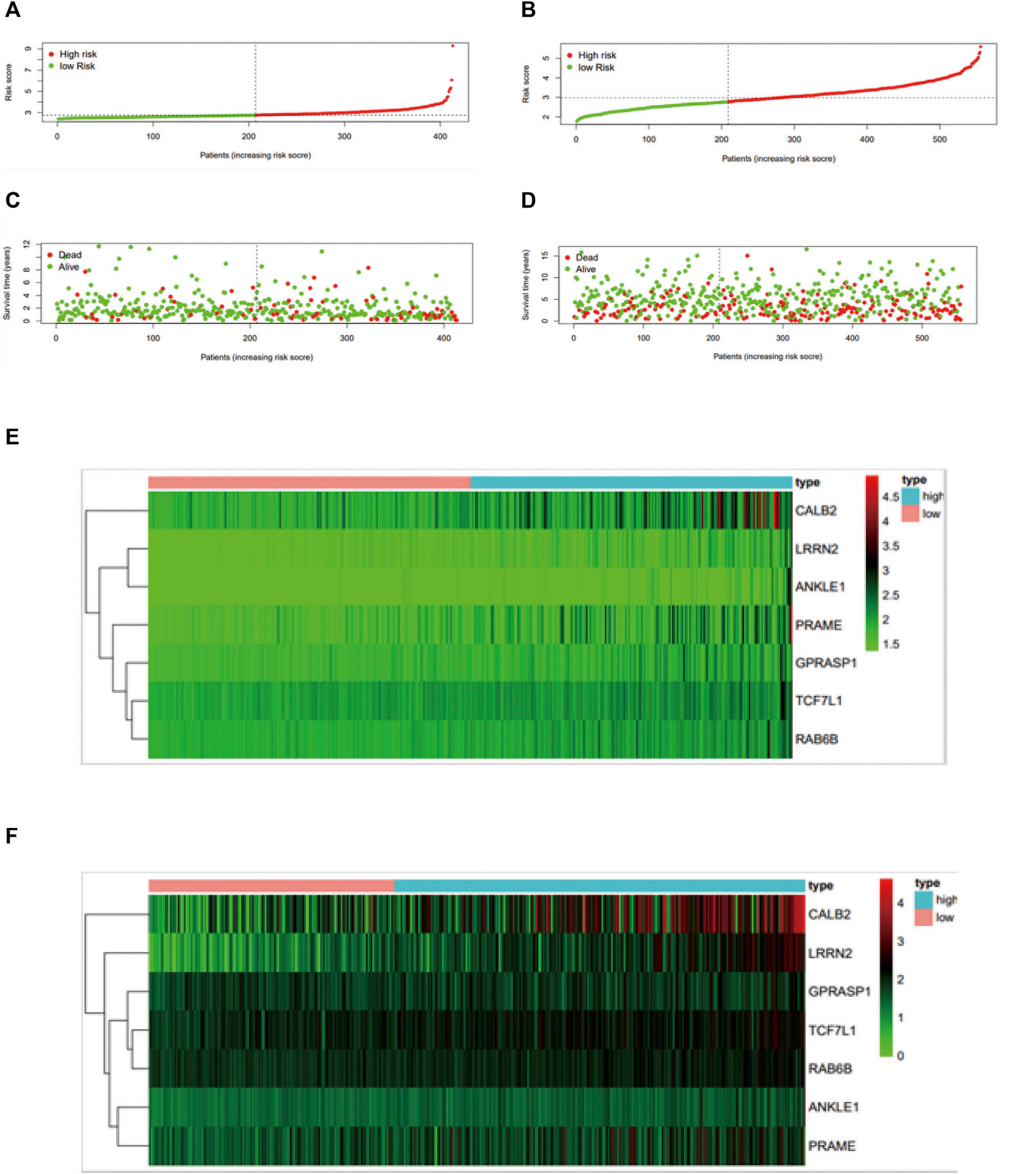
Construction and validation of the mtDNA-related prognostic signature. Assessment of LASSO regression analysis of 475 DGEs is shown here. **(A)** Risk score, **(C)** survival status, and **(E)** profiles of mtDNA-associated gene expression. The value of the mtDNA-related prognostic signature was validated in GSE39582 by the **(B)** risk score, **(D)** survival status, and **(F)** mtDNA-related gene expression profiles, and GSE39582 was displayed in Figure 4.

**FIGURE 4:**
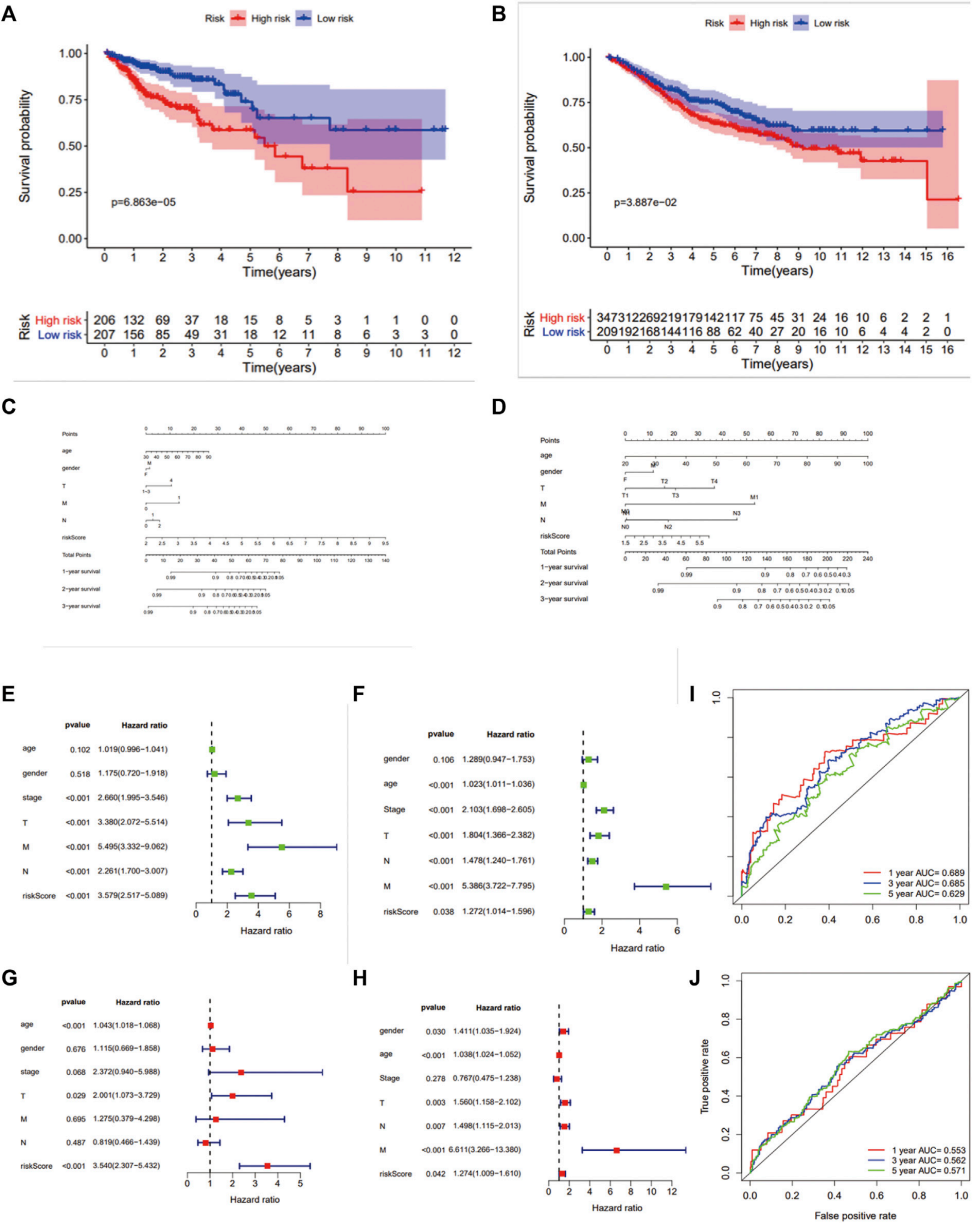
Survival analysis and ROC analysis of the 7-gene prognostic signature. Kaplan–Meier survival curves of OS **(A,B)**, OS predictive nomogram **(C,D)**, univariate **(E,F)** and multivariate Cox proportional hazards regression **(G,H)** and ROC curves **(I,J)** in patients with colon carcinoma in TCGA and GSE39582.

Colon cancer patients in TCGA were split into a high‐risk group and a low‐risk group by the median risk score and then validated in GSE39582 dataset (Figures 3A–F). As a result, the overall survival time of patients in the low‐risk group was significantly longer than that of the high‐risk group (*n* = 413, HR = 2.5,*p* < 0.0001), which was also validated in GSE39582 (*n* = 550,HR = 1.4, *p* < 0.05) (Figures 4A,B). The distribution of risk scores with age, gender, stage, pathologic stage(T), pathologic lymph node status, and metastasis were also analyzed by univariate and multivariate cox proportional hazards regression in the training dataset and validated dataset (Figures 4C–J).

### ESTIMATE Evaluation and Immune Infiltration Analysis

**FIGURE 5:**
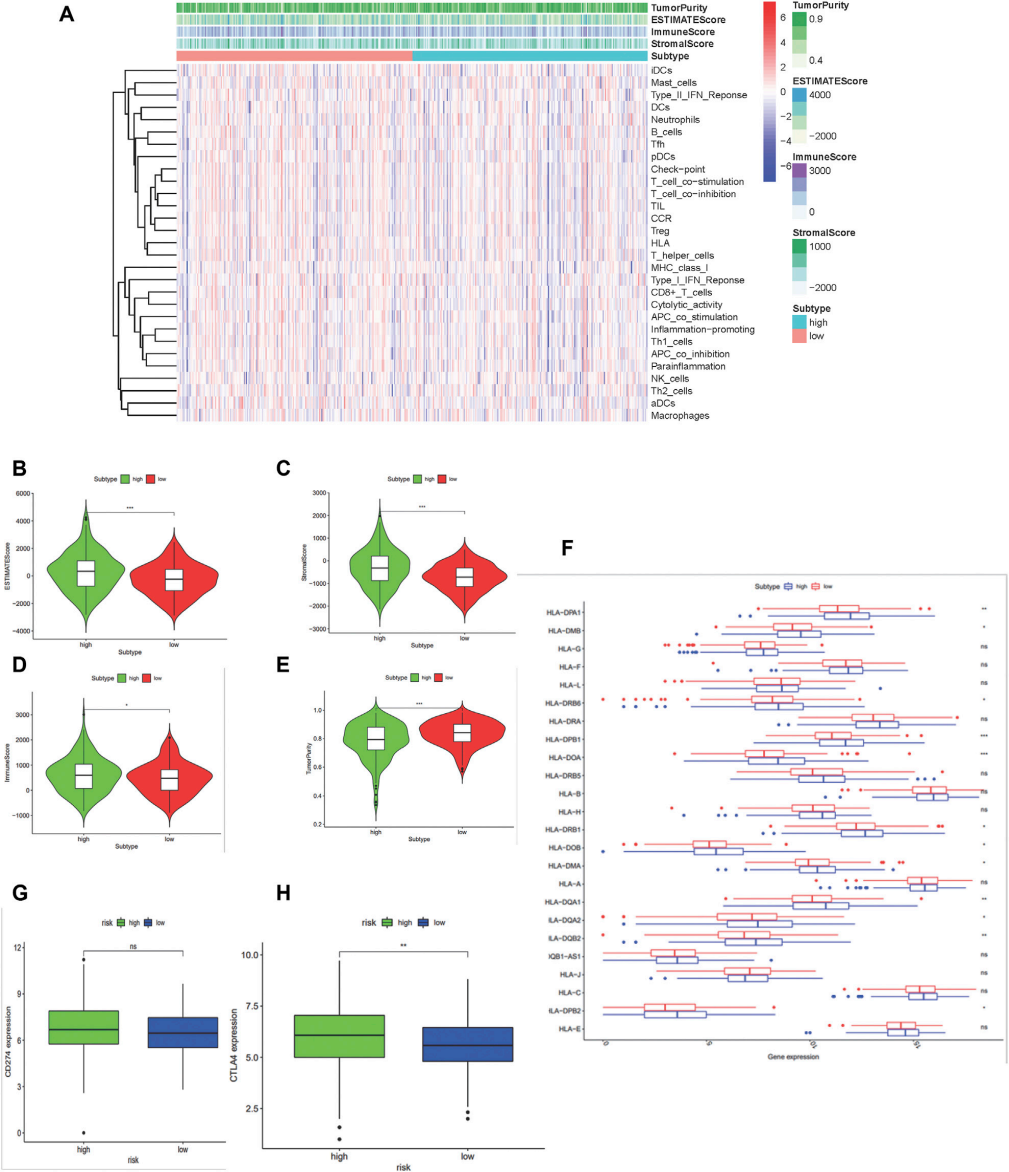
Construction and certification of mtDNA-related patients groups in colon carcinoma samples. **(A)** Binary risk group constructed by LASSO regression analysis of 475 DGEs and further validated using the ESTIMATE algorithm. **(B–E)** Differences of scores calculated by the ESTIMATE between the high-risk and low-risk group. **(F)** Boxplot shows the expression of HLA family genes. **(G–H)** Expressions of PD-L1 (CD274) and CTLA4. (*:*p* < 0.05 **:*p* < 0.01 ***: *p* < 0.001).

Recent studies suggest that the reprogramming of tumor metabolism led to local immunosuppression in the tumor microenvironment. An integrated bioinformatic analysis was applied to evaluate the differences of immune infiltration between the two groups with high- and low-risk scores established previously. As a result (Figure 5), the high-risk score group shows a higher ESTIMATE (*p* < 0.001), immune (*p* < 0.05), and stromal scores (*p* < 0.001) with a lower tumor purity (*p* < 0.001), which indicated that these patients with high risk scores may have strong infiltration of immune cells and stromal cells. Patients in the high-risk score group had a low-level expression in 12 HLA genes of the whole 24 HLA families and a higher expression of CTLA4 (*p* < 0.01) but no difference in the PDL1 expression.

**FIGURE 6:**
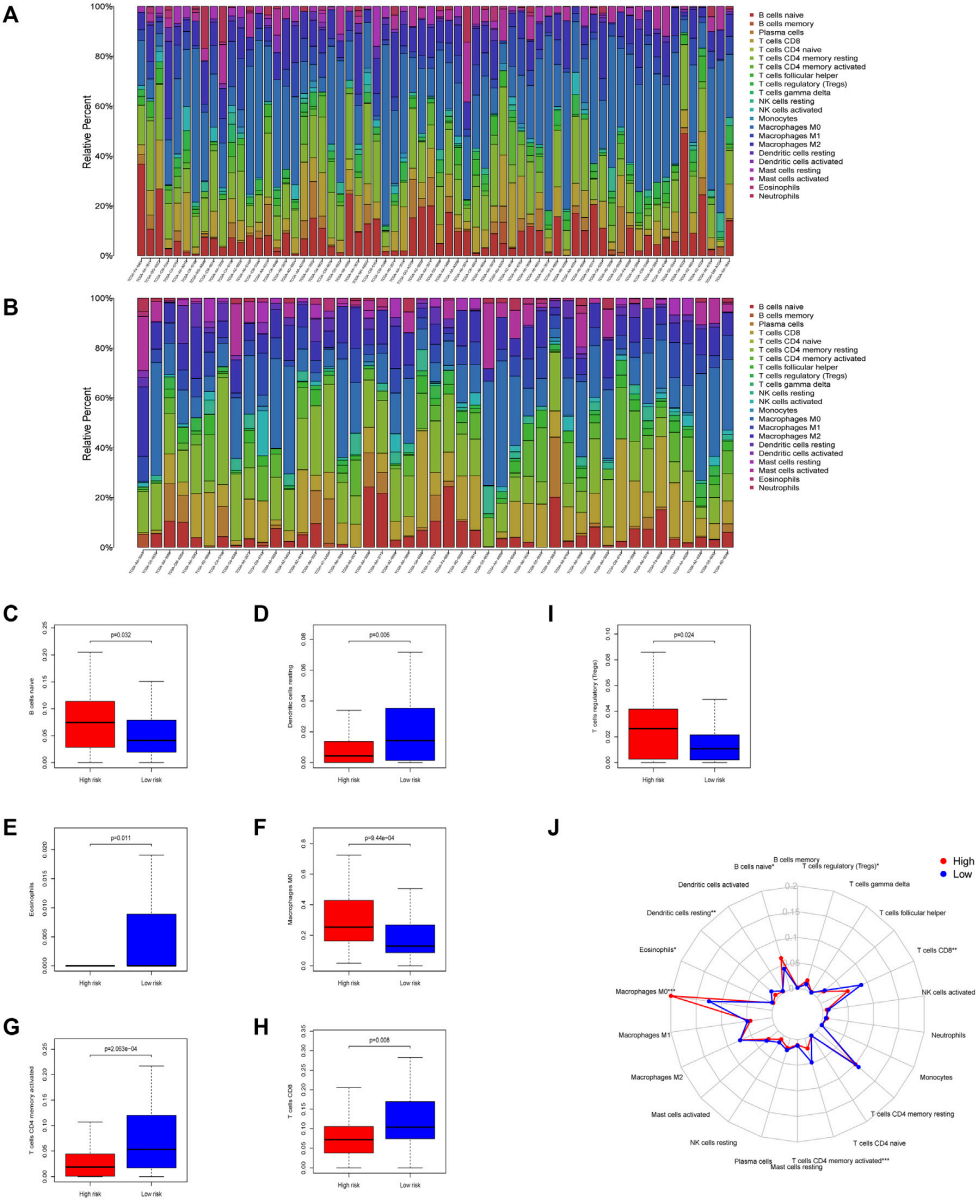
Immune infiltration conditions between the two mtDNA-related groups in colon carcinoma samples. Immune infiltration of high- **(A)** and low-**(B)** risk group calculated using the CIBERSORT algorithm was shown in the two barplot, respectively. **(C–I)** Differences of tumor-infiltrating immune cells during the two groups. Seven subtypes of tumor-infiltrating immune cells between the high-risk and low-risk group showed significant differences (*p* <0.05). **(J)** Radar plot shows the whole survey of CIBERSORT analysis (*:*p* < 0.05 **:*p* < 0.01 ***: and *p* < 0.001).

Compared to the low-risk group, CIBERSORT displayed different immune cell infiltrations in the high-risk group: downregulated dendritic cells (*p* = 0.006), CD8^+^ T cells (*p* = 0.008), and CD4^+^ activated memory T cells (*p* <0.0001), while upregulated naïve B cells (*p* = 0.032), M0 macrophages (*p* <0.0001), T regulatory cells (*p* = 0.024), and eosinophils (*p* = 0.011). These results suggested that patients with the high-risk score might benefit less from immunotherapy, which showed the value of the 7-gene prognostic signature in predicting the efficacy of immune checkpoint inhibitor immunotherapy. Above results are shown in Figure 6

### CD8^+^ T-Cell Infiltration and Mitochondrial Copy Number Variation

**FIGURE 7:**
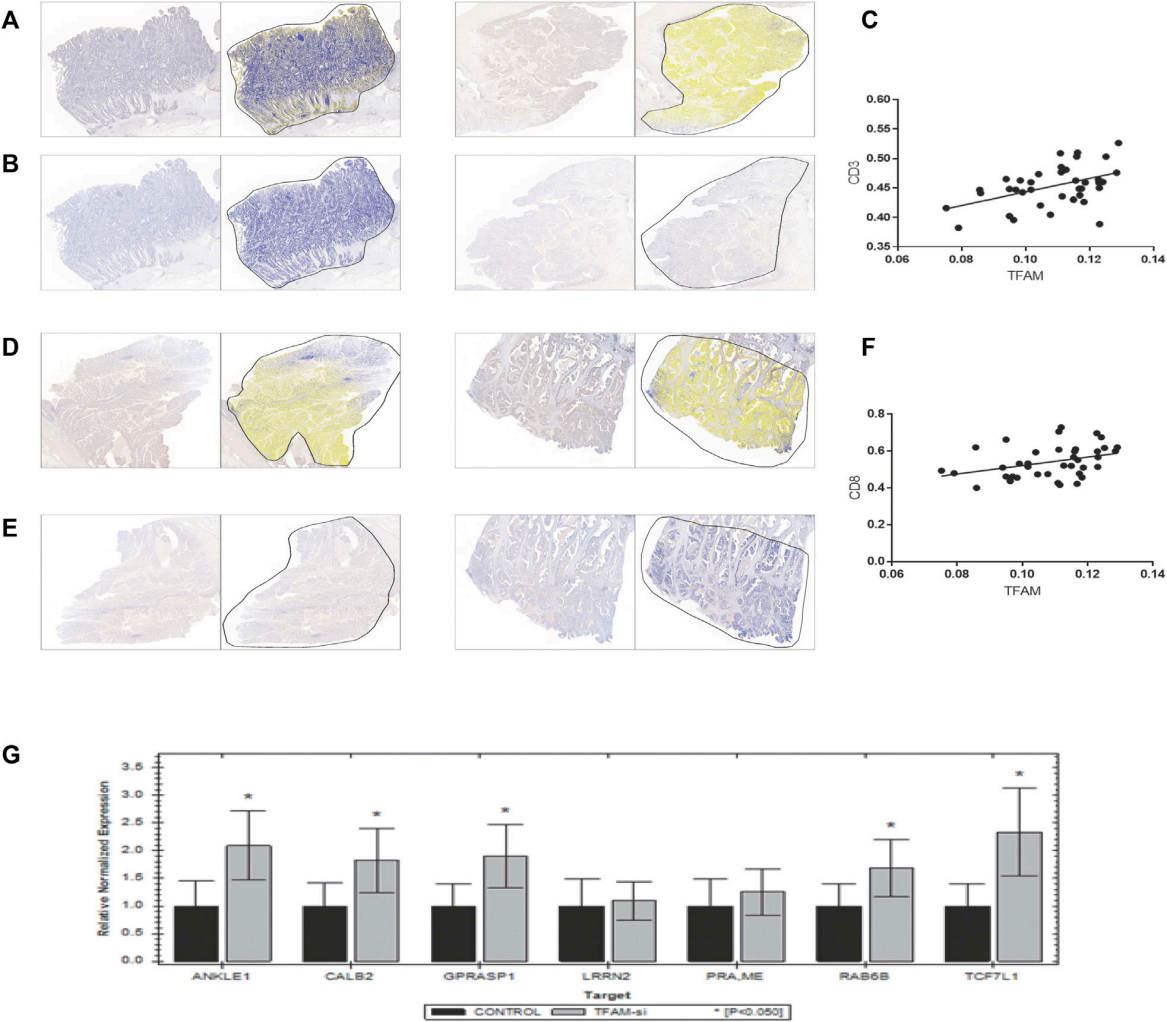
Verification of the correlation between T-cell infiltration and mitochondrial copy number. Results of immunohistochemistry of 39 colon patients are shown here. **(A,B,D,E)** were paired IHC tissues. As shown in results, tissues with a higher TFAM expression [left of **(A,B,D,E)**] have a stronger CD3 [right of **(A,B)**] and CD8 [right of **(D,E)**] expression. **(C,F)** shows positive correlation relationship of CD3 and CD8 with the TFAM expression (*p* = 0.0031 and 0.0237, respectively). **(G)** Results of the mRNA expression of seven signature genes in TFAM-knockdown SW480 cells by qPCR.

As previously mentioned, our findings had demonstrated that patients with lower mtDNA content relatively were less immune cell infiltrated, especially CD8^+^ T cells indicated using the ESTIMATE algorithm. To investigate whether mtDNA depletion was associated with a significant decrease of T-cell infiltration, we examined the abundance of the mitochondrial biomarker TFAM and T-cell infiltrations by CD3 and CD8 by immunohistochemistry (IHC) in the pathological section of colon cancer carcinoma in Zhongnan Hospital (Figures 7A,B,D,E). As a result, we found a significant positive relationship (*p* = 0.0031 and 0.0237, respectively) between CD3^+^ and CD8^+^ T cells and the expression of the mitochondrial biomarker TFAM (Figures 7C,F).

TFAM is critical for mitochondrial DNA replication, transcription, and stability, which has been confirmed to be related with poor prognosis in several cancers (Toki et al., 2010; Yoshida et al., 2011; Kurita et al., 2012; Kunkel et al., 2016). To investigate whether TFAM expression could be responsible for the decrease of T-cell infiltration in the mtDNA content-reduced condition, TFAM expression was knocked down by the TFAM siRNA lentivirus vector to knockdown in SW480 cells. Furthermore, five genes of the seven signature genes were significantly upregulated in TFAM-knockdown cells by qPCR (Figure 7G).

## Discussion

As per our results, patients with colon adenocarcinoma were split into two different prognostic groups based on the risk scores calculated according to the expression of mtDNA content reduction-related signature genes in TCGA. Although previous studies have demonstrated that mtDNA content reduction played a vital role in tumor initiation, progression, and drug resistance in colon adenocarcinoma, few studies investigated the relationship of mtDNA content and immune infiltration (Buckner et al., 2014; Wei et al., 2018; Chen et al., 2020). It has been commonly acknowledged that mitochondrial DNA content reduction induced tumorigenesis, metabolic reprogram, and biological alterations, which influenced the immunocyte distribution (Lee et al., 2005; Lee and John, 2015; Mou et al., 2018). Hence, we investigated the transcriptome profiling of EtBr-treated NCM460 (human immortalized colon cell line) to assess the biological alterations induced by mtDNA content reduction.

Among the whole 2,075 DEGs, there were 794 upregulated genes and 1281 downregulated genes. All these DEGs were evaluated by GO term enrichment and KEGG pathway analysis. As the results of GO enrichment analysis, most of these terms were associated with metabolic reprogramming which was one of the hallmarks in tumorigenesis (Srinivasan et al., 2017). Furthermore, the GO and KEGG analyses also proved that alternations induced by mtDNA content reduction were mainly proficient in the metabolic pathways in COAD, which deserves further exploration.

From the 2,075 mtDNA-related DEGs, 475 DEGs were selected using the Venn diagram of prognostic genes in TCGA datasets. A total of eight clusters were generated in MCODE. MCODE 1 was the most notable cluster among them. The 11 genes in MCODE1 (*CCR10*, *CXCL16*, *CXCL2*, *CXCL3*, *CXCL8*, *GPER1*, *NMU*, *SAA1*, *BDKRB2*, *CCL20*, and *CCL5*) were associated with cell chemotaxis, which induced the directional migration of cells including cancer cells and immune cells (Buckner et al., 2014; AbdelMageed et al., 2019; Chen et al., 2019; Zhao Q. Q. et al., 2020). The findings indicated that the defected mtDNA content in cancer cells might be associated with tumor progression and immune cell infiltrations in COAD.

Finally, a mtDNA content-related 7-gene signature was identified according to prognostic genes from colon adenocarcinoma transcriptome data. The efficacy of the risk scores and the integrated nomograms to predict the outcome of patients was evaluated in TCGA dataset (COAD) and validated in GSE39582 datasets. Therefore, our mtDNA content-related signature risk scores maybe a prognostic biomarker in colon adenocarcinoma.

The question followed was why the mtDNA content-related signature played such roles in overall survival. As indicated in results, the 7-gene prognostic signature showed significant differences of HLA family genes and immune checkpoint expression between two groups, which played critical roles in the antitumor immune system (Fridman et al., 2012; McGranahan et al., 2017; Topalian et al., 2020). In total, 12 HLA genes were significantly downregulated among the whole 24 HLA families in patients with high-risk scores, which indicated that these patients may benefit less from immunotherapy since immune evasion. We investigated the expression of major immune checkpoints PDL1, CTLA4, LAG3, and PDCD1. Regrettably, there was no significant difference in PDL1, LAG3, and PDCD1. However, the high-risk score group had a higher expression of CTLA4, which predominantly inhibits T-cell activation and immune response (Fiegle et al., 2019). CTLA-4 blockade was shown to inhibit tumor progression by upregulating effector T-cell activity and suppress regulatory T cells (Tregs), which suggest that the patients with high-risk score of the 7-gene prognostic signature might benefit less from CTLA-4 blockade therapy. Thus, it also demonstrated the value of the 7-gene prognostic signature in immunotherapy.

In this study, seven genes (*LRRN2*, *ANKLE1*, *GPRASP1*, *PRAME*, *TCF7L1*, *RAB6B*, and *CALB2*) were identified. *LRRN2* was the first to be reported upregulated in glioma and was related to cell adhesion and signal transduction (Almeida et al., 1998). *ANKLE1* was known as a new hotspot for the predisposition of breast cancer, which played a vital role in DNA damage response and DNA repair (Bakshi et al., 2020). *GPRASP1* was mainly localized in the cell cytoplasm and could translocate to the nucleus, which indicates it may be involved in transcription regulation and tumorigenesis by retrograde signaling (Abu-Helo and Simonin, 2010). *PRAME* is considered as a repressor of retinoic receptor, which is recognized by cytolytic T lymphocytes (Xu et al., 2020). *TCF7L1* encodes T-cell family factors and regulates cell senescence *via* the Wnt/*β*-catenin signaling pathway (Shan et al., 2019). *Rab6B*, whose family member affects the regulation of intracellular transport routes, has been known as an oncogene in colon cancer (Zhao L. et al., 2020). Moreover, it has been convinced that *CALB2* is associated with apoptosis, ECM, and poor clinical outcomes *via* the mitochondrial pathway (Stevenson et al., 2011; Ojasalu et al., 2020). In brief, the role of the seven genes in colon cancer and mitochondrial-associated tumorigenesis also need further exploration, especially *PRAME* and *TCF7L1*.

On the other hand, the high-risk group had a higher ESTIMATE, immune, and stromal scores with lower tumor purity, which indicated the patients with high-risk scores might have more immune infiltration. Previous studies proved that reprogramming of the tumor immune microenvironment was a valuable prognostic signature, such as a lack of T cells, microphage phenotype, the number of B cells, CD4^+^ T cells, CD8^+^ T cells, DC cells, and the ablation of eosinophils (Shankaran et al., 2001; Galon et al., 2006; Bindea et al., 2013; Herrera et al., 2013; Arnold et al., 2020; Petitprez et al., 2020). The abundance of CD8^+^, activated CD4^+^ memory T cells, and DC cells was downregulated, while M0 microphages, B naïve cells, and Tregs were \ significantly upregulated in the high-risk group, which showed a pro-tumor tendency. M0 cells were considered as another type of TAM or an incompletely differentiated M2 and strongly associated with the poor outcome in many different types of cancers (Yang et al., 2019; Zhang et al., 2020). It was fairly controversial for the roles of tumor-infiltrating B cells, which was both tumor-promoting and tumor-suppressing (Martinet et al., 2012; Dieu-Nosjean et al., 2014; Colbeck et al., 2017; Sautès-Fridman et al., 2019). As is known, Tregs depletion promotes tumor growth and tumor immune evasion (Wei et al., 2004). CD4^+^ and CD8^+^ T-cell responses play a key role in the process of malignant cell elimination (Ostroumov et al., 2018). B naïve cells had been confirmed as a biomarker to predict the immunotherapy response (Petitprez et al., 2020). In tissues from colon cancer patients, less CD3^+^CD8^+^ T cells and the higher expression of the mitochondrial biomarker TFAM were found. It could be exclaimed that the alterations of the gene expression induced by mtDNA content defects brought about TME reprogramming, which indicated the potential of our risk score to forecast the effect of immunotherapy. Taken together, the 7-gene signature might work in the process of carcinogenesis, proliferation, and cancer immune evasion. Further exploration of these findings was required.

In conclusion, by multiple bioinformatics analysis, the hub genes of the DEGs intersected from TCGA and the transcriptome data of EB-treated NCM460 were screened, and the 7-gene prognostic signature was established in colon cancer initiation and progression. The predicting efficacy of the 7-gene prognostic signature could be attributed to the variations of immune infiltration and immune checkpoint in the tumor microenvironment. Our finding provides novel insights into the roles of mitochondrial DNA content reduction in TME reprogramming.

## Data Availability

The datasets presented in this study can be found in online repositories. The names of the repository/repositories and accession number(s) can be found in the article/Supplementary Material.

## Glossary

**ANKLE1**: ankyrin repeat and LEM domain containing 1
**AUC**: area under the curve
BP: biological process
**CALB2**: calbindin 2
**CC**: cellular composition
**CIBERSORT**: Cell-type Identification by Estimating Relative Subsets of RNA Transcripts HR Hazard ratio
**CTLA4**: cytotoxic T lymphocyte-associated antigen-4
**DEGs**: differentially expressed genes
**ESTIMATE**: Estimation of Stromal and Immune cells in Malignant Tumor tissues using the Expression data
**FDR**: false discovery rate
**FPKM**: fragments per kilobase per million
**GEO**: Gene Expression Omnibus
**GO**: gene ontology analysis
**GPRASP1**: G protein-coupled receptor associated sorting protein 1
**HLA**: human leukocyte antigen
**HTSeq**: high-throughput sequence
**ICIs**: immune checkpoint inhibitors
**KEGG**: Kyoto Encyclopedia of Genes and Genomes
**LASSO**: least absolute shrinkage and selection operator
**LRRN2**: leucine rich repeat neuronal 2
**MCODE**: Molecular Complex Detection
**MF**: molecular functions
**mtDNA**: mitochondrial deoxyribonucleic acid
**PD-L1**: programmed death-ligand 1
**PPI**: protein–protein interaction
**PRAME**: PReferentially expressed Antigen in MElanoma
**OS**: overall survival
**RAB6B**: member RAS oncogene family 6B
**ROC**: receiver operating characteristic
**STRING**: Search Tool for the Retrieval of Interacting Genes database
**TCF7L1**: transcription factor 7-like 1
**TFAM**: transcription factor A, Mitochondrial; TCGA The Cancer Genome Atlas
**TIICs**: tumor-infiltrating immune cell
**TME**: tumor microenvironment
